# Announcement: The First International Conference on Trace Element Speciaton in Biomedical, Nutritional and Environmental Sciences

**DOI:** 10.1155/MBD.1997.243

**Published:** 1997


### THE FIRST INTERNATIONAL CONFERENCE ON TRACE ELEMENT SPECIATON IN BIOMEDICAL, NUTRITIONAL AND ENVIRONMENTAL SCIENCES

May 4 -7, 1998

GSF - Forschungszentrum
Neuherberg/Munchen, Germany

Six International Workshops on Trace Element Analytical Chemistry in Medicine and Biology have been organized in the years 1980 - 1991 in the GSF Research Centre. More than 5 years are past now and the topics in Trace Analytical Chemistry have significantly changed in the last decade. No longer are the concentration values in a certain matrix in the foreground of our interest. Instead of this, knowledge about the species like oxidation state, binding forms, etc becomes more and more of interest for a better understanding of the role of all trace elements in the biomedical, nutritional as well as the environmental field. Speciation becomes a new challenge to the analytical chemist, because nearly all wellknown steps in classical trace element analysis change. Furthermore, Speciation has not been a central focus of symposia concerning trace element studies. Our intention now is to focus on these broad, new and sometimes diffuse activities and to create a forum for open discussions about very new and perhaps not fully established ffindings Speciation is an often used term in the literature, but not generally well defined. Some different definitions, outgoing from very different points of view, are available. Mainly derived from the analytical method used, the species are defined from a functional or operational standpoint or as a specific chemical form (ligand) or oxidation state. The challenge for speciation research should be the latter one, that is identification and quantification of the species.

### Invitation and Call for Papers

The Scientific Committee extends an invitation to all scientists involved and/or interested in "Speciation Analysis". A major goal of the Conference is to facilitate discussions about all aspects of this very new analytical field, including analytical chemistry, biochemistry, essentiality and nutrition, medical uses and environmental sciences.

### The Scientific Programme

The Conference Programme will comprise three days of oral presentations, posters and extended time for discussion. Oral presentation (invited or submitted) will be sixty or twenty minutes in duration including the specific discussion. The tradition established in the six Workshops of assigning a prominent and central role to poster presentations will continue. Consequently, special poster discussions in the auditorium will be organized, where the authors will have the possibility to present orally the highlights of their poster. Round table discussions will be organized on special topics. (The organizing committee would, at this stage, welcome suggestions for special topics.) On the fourth day, short courses related to highly significant methods, problems and applications will be offered.
The main topics of the First International Conference will be (For every topic the „state of the art" will be presented by a plenary lecturer):

Separation and hyphenated techniques
Quality assurance and reference materials in speciation analysis
Speciation in the biomedical field
Speciation in the environmental field
Speciation in the nutritional field
Speciation in occupational health
Speciation and legislation

### Conference Proceedings

As for the previous six Workshops, all papers presented as posters or lectures may be submitted as full papers for publication, now in the "Fresenius' Journal of Analytical Chemistry" subject to the normal review procedure of the journal.

### Conference Location

The venue of the Conference is the GSF-Research Center (Auditorium) in Neuherberg near Munich (Germany).

### Registration Fee

The registration fee per delegate is DM 650.-- and includes one free copy of the Proceedings, the Workshop-Dinner and Coffee-Breaks. The registration fee for students is DM 350.--. Single day registration (without Proceedings and Dinner) is DM 250.--.The fee for one course (half a day) will be DM 150.--For accompanying persons DM 150.-- are quoted (includes Conference Dinner and Social Programme).

### Secretariat

First Speciation Conference
GSF- Forschungszentrum
Congress Service Postfach 1129
D- 85758 Oberschleissheim Germany

*General and Registration:*
c/o Ulla Schrodel
Tel.:089/3187-3030 (-2669)
Fax.:089/3187-3362

*Scientific:*
c/o Dr. B. Michalke
Tel.: 089/3187-4206
Fax.: 089/3187-3348

c/o Prof.Dr. P. Schramel GSF -
Tel.: 089/3187-4062
Fax.: 089/3187-3348

### Special Announcement

Following the Conference, the 5th Conference on Metal Ions in Medicine and Biology will be organized from May 8-10.,1998.
Information on this Conference can be obtained from
Dr. V. Negretti de Bratter,
Hahn Meitner Institut Berlin
Glienicker Str. 100
D-14109 Berlin Germany
FAX: +49 (030) 8062 2781
E-mail: negretti hrni.de
WWW: http://www.hmi.de/bereiche/N/NG/Events.html

A possible reduction of the conference fees for participants of both conferences will be indicated in the 2nd Circular.

